# Dental Calendar

**Published:** 1883-08

**Authors:** 


### DENTAL CALENDAR.

We have undertaken the task of publishing a list of the Dental Societies of America, with the time of their regular meetings and the names of the Presidents and Secretaries. Such a register can but prove a great convenience to dentists, but to secure all its advantages it should be complete, and kept correct up to date. We shall do our best to make it such, but we must necessarily depend upon the kindness of friends for success. This month we publish a list of all the societies that we have been able to obtain, but the roll is far from being complete. We hope to add to it, until any one who desires knowledge of any dental society will need only to look in his copy of the INDEPENDENT PRACTITIONER for the information. The register will, however, be only misleading, unless we are kept fully posted concerning the changes made. The secretaries or members of every dental society in the United States are therefore earnestly requested to communicate with Dr. O. E. Hill, 160 Clinton Street, Brooklyn, N. Y., who will take charge of this department, or to send the desired information directly to the editor.

We shall be very glad to receive notices of coming meetings, and they will always have place in "Current News and Opinions."

